# The Swine-plague (Swinepest) in Austria

**Published:** 1896-05

**Authors:** 


THE SWINE-PLAGUE (SWINEPEST) IN AUSTRIA. Abstracted from the report of Prof. Hugo Schindelka, published in the *Thierärztliches Centralblatt* (*concluded from page* 287). As in the case of other epidemics, so in the swine-plague, several stages in the course of the disease can be observed. In regard to the length of the stage of incubation the reports received differ widely. If we take all reports into consideration, we must assume that the time of incubation lasts from forty-eight hours to four weeks. The majority of the observations, however, place the time of incubation at about fourteen days, three reports give nineteen to twenty-one days, and one a duration of four weeks for this stage.

The breed, age, shape of the body, and condition do not appear to influence the stage of incubation, since the reports in this regard are either entirely wanting or very contradictory. At first it appeared to me as if the duration of the incubation stage was shorter in the case of finely bred English hogs than in those of Hungarian, Polish, or German origin; but after extended observations I find that this is not the case. I must, however, side with those who accept a longer duration for the stage of incubation. I believe, therefore, in accordance with the observations so far made, that the period of incubation in this disease lasts from two to over three weeks. A shorter or longer period of incubation may be considered exceptional. I do not wish here to deny the correctness of such assertions, but feel compelled to call attention to the fact that the belief in an incubation stage of one to six days can rest only upon an error which is pardonable, as the statements of hog-owners in this matter are not to be depended upon, especially when made by people who are not acquainted with the symptoms of the disease.

The clinical course of the disease was in more than one respect a varied one. Aside from the disturbances in the general condition, always present, in a number of cases the organs of respiration were extensively affected; in other cases the digestive tract was especially attacked by the disease; and in a third group of cases the organs of respiration and those of digestion were affected to the same extent and simultaneously. Many cases presented changes in the organs of sight, in the skin, and in the urinary organs.

But that is not all: besides the differences mentioned in localization, differences occurred in the time of onset of the symptoms, so that the cases may be distinguished as peracute, acute, subacute, and chronic.

From this the manifold forms of the disease can be explained, inasmuch as the most varied combinations of the differences mentioned occurred in the localization, the course of the disease, and the order in which the phenomena showed themselves. The affection, as a rule, first shows itself in severe disturbances in the general condition; in the majority of cases the swine are affected suddenly, a shaking chill being usually the precursor. The animals creep to a corner, avoid movement, and appear from the beginning without interest; they are languid in their movements, a symptom which shows itself plainly when they are stirred from their beds and made to move quickly. All movement seems to cause pain, which causes marked lameness, now of one, now of the other extremity. When stirred from bed they move slowly around with their back bowed, the head and neck stretched out, putting the legs, which are held stiff, down very carefully. They allow themselves to be caught without resistance. In most cases, after some days the condition seems to grow better, either permanently or for only a short time. In the cases which show a rapid progress of the disease the hinder parts of the animal are very unsteady, incapable of rapid movement, and often give way together. In one or two cases epileptic attacks have been observed.

The disease begins with fever, which becomes very high in acute cases. Shaking chills often appear, not only in the first onset of the disease, but in the more advanced stages, and in chronic cases are sometimes the prelude to a fresh attack of fever. The chills are sometimes so severe that the whole animal is moved to and fro on the bed. But it is to be remarked that chills, although usually present, are sometimes absent. The course of the fever is in all cases highly atypic.

The pathological changes in the organs of respiration show themselves in a more or less severe dyspnœa, in which the depth of the inspiration was not so noticeable as the fact that the number of respirations increased almost incredibly. I could often count from 100 to 120 respirations to the minute. This was the case especially at the beginning of the disease, at a time when neither symptoms of bronchitis nor of other changes could be detected which pointed to a diminution of the respiratory surfaces, as is the case in pneumonia and pleuritis. It is further worth mentioning that this dyspnœa in the peracute and acute cases was permanent, and only relaxed for a short time in exceptional cases, and then showed the character of paroxysms.

In many cases the disease was accompanied by coughing, which came on mostly in spells. It was sometimes dry and painful, at other times accompanied with excretion, and in three advanced cases, in which pneumonic changes were present, it showed itself as a continuous short cough.

Finally, I may add, that in not a few cases pleural friction sounds could easily be detected. Running from the nose was often present, and in such abundance that the nasal septum was covered to a great extent with a firm, very sticky, yellowish-brown incrustation. The secretion, at first serous, assumes a mucous or muco-purulent consistency, and is similar to the excretion often seen in cases of epilepsy in the dog. According to Mr. Zaubek, about 50 per cent. of all cases show excretion.

Besides this, bleeding at the nose was also observed. Mr. Postolka saw this first in Vienna among swine of English breed, and, if I am not mistaken, as the initial symptom. We thought that this phenomenon was peculiar to the cases which take an acute or peracute course, but were soon convinced that this was not always the case, and we afterward saw a case with this symptom where the disease continued for a considerable length of time.

When I add that the changes mentioned are mostly associated with disturbances in the digestive tract, it is all that I can briefly communicate in regard to this at this time. It might be mentioned, however, that the cases in which the organs of respiration are affected admit of only a very unfavorable prognosis, inasmuch as I cannot cite a single case among the many that came to my notice where a cure was effected. Some cases which took a chronic course recovered, apparently in spite of permanent changes in the lungs, and attained even a certain degree of corpulency. Disturbances in the digestive tract showed themselves clinically in poor appetite, in great thirst, in vomiting, diarrhœa, and sometimes in constipation. These disturbances appeared simultaneously with those in the general condition. Feeding was interfered with and continued during the course of the disease until the appetite failed entirely. The thirst was always very severe, and the observation is worthy of mention that the sick hogs preferred filthy water which had been befouled with urine and excrement to pure water. The mucous membrane of the mouth was dry from the beginning and the tongue cracked. Besides this, vomiting is to be mentioned among the initial symptoms observed in a great number of the sick swine. Often bright yellow or brownish-yellow matter, mostly in tough and shiny masses, was emitted. The act of vomiting was repeated several times, as a rule, and often without especially severe effort. I must call attention to the fact, however, that vomiting is not one of the constant symptoms of the disease, but is wanting in numerous cases. In the reports this symptom is seldom mentioned. The communication of Herr Dobes is interesting, inasmuch as he has observed among other gastric symptoms meteorism (flatulent distention of the abdomen).

Of the other symptoms in the digestive tract diarrhœa and costiveness are to be mentioned first. Often in the further course of the disease diarrhœa follows constipation, or the one alternates with the other in chronic cases. In young animals diarrhœa was observed oftener, in older ones constipation. I believe the exhausting diarrhœa, together with fever, to be the cause of the emaciation and the great loss of strength which follow very rapidly. The observation could sometimes be made that a well-fed animal would fall away to a skeleton in a very few days. With the initial symptoms of this disease, and sometimes even before these, especially in young animals or acute cases, diarrhœa began. The excretions had a greenish-yellow, brownish-yellow, or bright yellow color, contained sometimes a mixture of blood, and had a slimy consistency and a fetid smell. In acute cases the diarrhœa remained constant. The evacuations were frequent, and before death apparently involuntary.

In another series of cases, and especially in those showing a prolonged course of the disease, constipation was observed from the beginning. The animals, as the owners stated, and we could verify, did not make manure for one or two days, then had an evacuation of solid excrement of the following consistency: strongly inspissated, solid dung was evacuated in the form of hard lumps of various size, the surfaces of which, in advanced stages, were covered with croup-like membranes showing little canals of blood. The color of the lumps was from a dark to a black-brown, very seldom clay-colored. This last color, as well as the absence of membranous fat and blood, were warranted a good prognosis.

The disturbances in the health which have been so far enumerated formed, so to speak, the commonest and constant condition, and appeared as a rule in conjunction with disturbances in the intestinal tract or with disturbances in the respiratory organs.

The other clinical changes which, as mentioned, affected the eyes, the skin, and the urinary organs, formed a complication which was not present in the majority of cases. I will now briefly speak of the changes in the organs of sight. Very frequently a severe conjunctivitis was present with photophobia. The conjunctivitis was marked in many cases with profuse secretion of a tough, slimy substance which caused adhesion of the lids. Now and again a peripheral clouding of the conjunctiva was seen, and a deeper affection of the organ of sight was not seldom observed. Several veterinarians were able to prove the existence of exudation in the front optical chamber, and Herr Niemetz mentions some cases of total blindness. Although almost all observers assert that the affection of the eye was very frequent, some going so far as to say that almost all swine showed a purulent conjunctivitis, nevertheless I must assert, in opposition, that in great numbers of cases, and in such as often take an acute or fatal course, that beyond aversion to light and a somewhat severe inflammation of the conjunctiva, no further affection of the organs of sight could be detected. I feel compelled to call attention to this in order that the diagnosis of the disease may not be made dependent upon the presence of disturbances in the eyes. In judging the different cases definite conclusions for the prognosis cannot be drawn from these changes.

Changes of an eczematous nature I have never observed, and in the light of my own experience I doubt their occurrence in this epidemic. I believe, likewise, that the necrosis of the skin as it has been observed here and there does not stand in direct connection with this disease, but I do not wish to deny that both processes may often occur at the same time. The occurrence of hemorrhages into the skin has been mentioned by several observers. In fact, this occurs very often in the form of petechiæ, vibices, and ecchymosis, less frequently as purpura hemorrhagica. These formations of blood are usually the endsymptoms, and are more prominent on dead than on living animals, especially after scalding.

The pathological changes in the urinary organs concern mostly the amount and nature of the urine. A diminution of the quantity of urine can be observed, and then a very decided excess of the same, and finally bloody urine. The first case could be brought about by the presence of profuse diarrhœa. Excessive urine is not a very uncommon phenomenon. I have been able to observe it several times at the beginning of the disease and once in its course. To what cause these symptoms should be attributed I do not know, as I had no opportunity to observe these cases further and none to examine the urine. They are probably connected with the affection in the kidneys. As far as bloody urine is concerned I have not had sufficient experience myself, nor do the reports give information of this. I saw such cases several times, but had no opportunity to follow them up. A few times I was present at the post-mortem examinations, which showed, among other symptoms of septicæmia hemorrhagica, infusions of blood in the kidneys. The animals so affected very probably showed bloody urine during life.

THE SWINE-PLAGUE IN AUSTRIA ON THE DECLINE. The swine-plague which has been raging in Austria during the past year is now steadily on the decline. Compared with the report of January 7, 1896, the number of districts afflicted show a decrease of 14, the number of places 86 less, and the number of farms 596 less. The epidemic is, according to the report of January 21st, still present in 51 districts, 131 places, and 1751 farms. The number of swine endangered amounts to 7499 head, of which 4608 are sick.

In Hungary, also, the pest has declined gradually, and, according to the report of January 22d, is still present in 36 districts, 12 cities, and 928 places.—TOSCANO'S REPORT in the *Thierärzt. Centralblatt*, February 15, 1896.

---

TEN per cent. trikresol soap will be found a useful medicament for those dry, scurfy skin-eruptions along the backs of cats, often resulting from imperfect assimilation of food, and incidental to old age. The soap moistened sufficiently to make a lather, and allowed to dry on, is a very satisfactory method of using it.

